# Fatigue Performance of a Step-Lap Joint under Tensile Load: A Numerical Study

**DOI:** 10.3390/polym15081949

**Published:** 2023-04-19

**Authors:** Murat Demiral, Ali Mamedov

**Affiliations:** College of Engineering and Technology, American University of the Middle East, Egaila 54200, Kuwait; ali.mamedov@aum.edu.kw

**Keywords:** step-lap joints, fatigue, tensile loading, Paris law, cohesive zone model

## Abstract

In many technical domains, adhesively bonded joints have been employed extensively. These joints perform poorly against peel stresses despite having good shear characteristics. A step-lap joint (SLJ) is one of the techniques used to reduce the peel stresses at the edges of the overlap area to avoid damages. In these joints, the butted laminations of each layer are successively offset in succeeding layers in the same direction. Bonded joints are subjected to cyclic loadings in addition to static loads. It is difficult to predict their fatigue life accurately; however, this information must be clarified to explain their failure characteristics. To this end, the fatigue response of an adhesively bonded step-lap joint subjected to tensile loading was investigated with the developed finite-element (FE) model. In the joint, toughened type DP 460 and A2024-T3 aluminium alloys were used for the adhesive layer and adherends, respectively. The cohesive zone model with static and fatigue damages were linked to each other and were used to represent the response of the adhesive layer. The model was implemented using an ABAQUS/Standard user-defined UMAT subroutine. Experiments found in the literature served as a basis for validating the numerical model. The fatigue performance of a step-lap joint for various configurations subjected to tensile loading was examined thoroughly.

## 1. Introduction

In recent years, adhesively bonded materials have become one of the most commonly used types of bonded materials in various industries, including the aerospace, automotive, and maritime industries. Their ability to bond similar and dissimilar materials along with satisfactory shear properties makes them so handy for industries seeking weight-to-strength ratio optimization [1]. Moreover, regularly bonded components have shown a weak performance when subjected to peeling stress [1,2]. Therefore, numerous groups of researchers have proposed different solutions, including the step-lap joint [3,4], scarf-lap joint [5,6], spew fillet [7], and composite patch [8,9] configurations to overcome this drawback.

Studies related to the step-lap joint are explained briefly in the next section. Mistry et al. [10] performed a detailed numerical analysis of bolted, riveted, bonded, and hybrid step-lap joints under tensile and bending stresses. Their studies revealed that a 2-21-2 mm long simple adhesive step-lap joint induced less stress and deformation compared to all other configurations, and it was the most suitable joint for components subjected to tensile and bending loads. To the contrary, one rivet joint induced the maximum amount of stress and was not suitable for tensile loading applications. Silva et al. [11] compared the performance of a single-lap joint and step-lap joints under tensile loading, and concluded that for joints that have an overlap length of 50 mm or more, step-lap joints performed better. However, the authors emphasized that for the final selection of the suitable joint, the performance of the step-lap joint had to be weighted against the ease of manufacturing the single-lap joint. To the contrary, Wang et al. [12] proposed an analytical model for the analysis of step-lap joints subjected to tensile stress. Using the proposed model, adhesive shear and peel stress distributions of the double-lap joint could be obtained in a closed form solution. Wang et al. [13] used a free vibration analysis to investigate the vibration characteristics and damping properties of the adhesive layer of single-lap and two-step-lap joints. The study revealed that the configuration parameters, such as the adhesive loss factor, height ratio of the steps, overlap area, adhesive thickness, and step number have a significant influence on the vibration behaviour of the joints. Sawa et al. [14] used a four-point bending test to characterize the behaviour of step-lap joints of dissimilar adherends. The researchers pointed out that dissimilar adherends performed poorly compared to similar adherends, and the pick value of the principle stress occurred at the butted edge of the adherend’s interface with a higher Young’s modulus.

From the publications presented above, it can be seen that studies on composite fatigue behaviour have evolved recently, mainly driven by their demand in the aerospace industry. A sufficient amount of research was performed on step-lap joints subjected to various static loading conditions. It has been shown that they do not only undergo static loading, but there are numerous applications in which these joints are subjected to cyclic loads. Limited research focusing on the fatigue performance of lap joints is available in the literature. Gavgali et al. [15] discussed that applying a three-step-lap onto the overlap area of single-lap joints subjected to tensile fatigue testing considerably increased the fatigue strength limit of the joint. Chowdhury et al. [16] evaluated the performance of different lap joint configurations of thick carbon fibre/epoxy laminates, including the step-lap joints under cyclic loading, and revealed that the hybrid joints have the greatest durability, followed by bolted joints, and finally by bonded joints. Recently, Ravi Chandran [17] presented a review on the fatigue of fibre-reinforced composites, their damage, and failure behaviours. Prakash et al. [18] presented research on the fatigue damage estimation of metals and fibre-reinforced composites. Godzimirski et al. [19] evaluated the fatigue behaviour and failure mechanisms of riveted lap joints of CFRP composites. Kang et al. [20] presented a probabilistic analysis of the fatigue life of fibre-reinforced composites based on the damage accumulation behaviour.

An accurate prediction of the fatigue life of the step-lap joints is essential for the proper characterization of their service life due to the fact that such loadings may result in failure, even at a small percentage of their static strengths. In none of the above studies were the failure characteristics of the step-lap joint under cyclic tensile loading thoroughly investigated. For this purpose, in this study, an advanced finite element model using the user-defined UMAT subroutine available in ABAQUS/Standard, was employed. The service life prediction, along with the crack initiation cycle (*N_i_*), the crack propagation rate (*da*/*dN*), and the failure cycle (*N_f_*) of the joints composed of the toughened type DP 460 and A2024-T3 aluminium alloys, as the adhesive layer and adherends, respectively, are investigated in this study. The cohesive zone model with static and fatigue damages that were linked to each other [21] was used to represent the response of the adhesive layer. Experiments from the literature were used as a basis for validating the numerical model. The fatigue performance of a step-lap joint for various configurations subjected to tensile loading was investigated in depth.

## 2. Numerical Modelling

### 2.1. Finite Element Modelling

**Figure 1:**
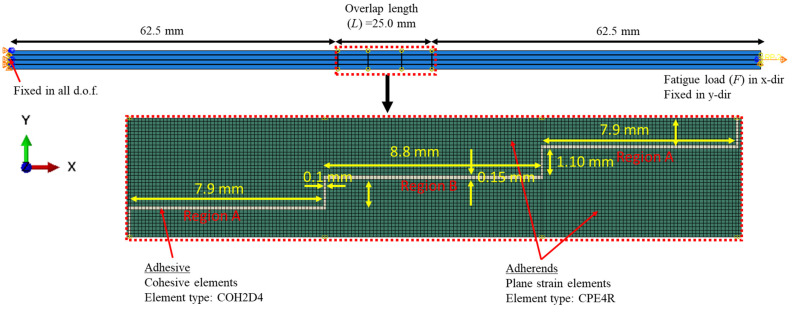
An FE model of an adhesively bonded three-step-lap joint under cyclic tensile loading.

A two-dimensional FE model of an adhesively bonded three-step-lap joint under cyclic tensile loading was developed via ABAQUS/Standard [22], as shown in Figure 1. As the model was validated with the experimental results in [15], its dimensions were selected in accordance with the ones in those experiments.

The behaviours of the adhesive layer and adherends were modeled using plane strain elements (CPE4R) and cohesive elements (COH2D4), respectively. Both of them are isotropic and homogenous materials, and hence are modelled as such in their section definitions in the model. A mesh convergency study was performed. Element sizes of 0.240 mm × 0.240 mm (coarsest), 0.120 mm × 0.120 mm, and 0.060 mm × 0.060 mm (finest) were considered for both the adhesive layer and the adherends. The von Mises stress value at the left-end of the element of the adhesive layer was calculated using a 9.0 kN tensile load applied for different mesh sizes, and then they were compared. This applied load was the maximum load applied within the static step beyond which the fatigue step continued (more details about this can be found later in this section). It was noted that the change in that stress was 8.9% and 3.5% when the mesh was changed from the coarsest mesh to the medium-sized mesh, and then from the medium-sized to the finest mesh. As a result, an average element size of 0.120 mm × 0.120 mm was chosen to be used, both in the adhesive layer and the adherends, as the respective result using this mesh was less than 5.0% when compared to that using the finest mesh. The adherends outside of the overlap region were meshed more coarsely when compared to those in the inside region with a bias ratio of 4 towards the overlapping area. Overall, 9762 CPE4R elements and 203 COH2D4 elements exist in the FE model, as shown in Figure 1.

The left-end side of the step-lap joint was fixed in all degrees of freedom. A kinematic coupling was defined on the opposite side, where the centre point was controlling this end surface. It was free to move only in the x-direction, where the tensile load was applied from this centre point. In the simulations, 9.0, 8.0, and 7.0 kN cycling load *F* values were used.

**Figure 2:**
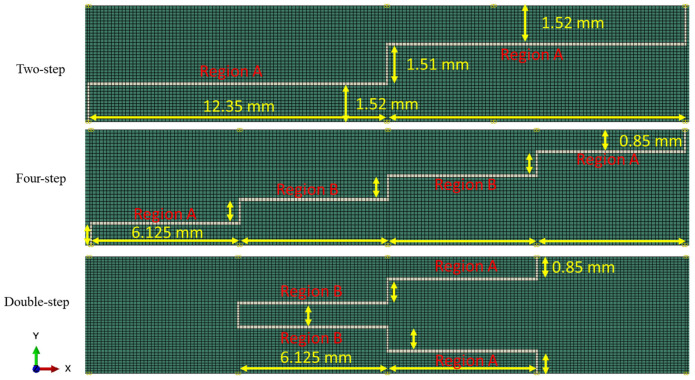
Geometric details of the two-step-, four-step-, and double-step-lap joints.

In this study, various step-lap joints with different numbers of steps and configurations were analyzed. Figure 2 shows their details. Namely, the two-step-, four-step-, and double-step-lap joints, on top of the three-step-lap joint, were compared and contrasted.

**Figure 3:**
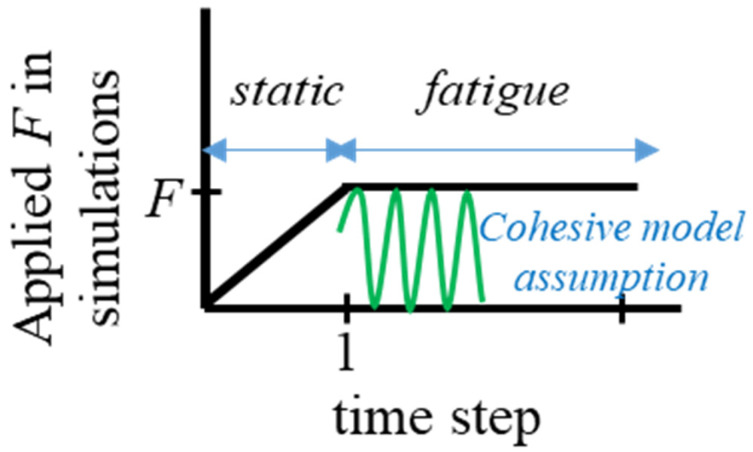
The variation of the tensile load applied to the step-lap joints.

ABAQUS’ elastic fully plastic constitutive equations were utilized to analyse the adherends’ behaviour. The behaviour of the adhesive layer was simulated using the fatigue damage model, that was created by degrading the bi-linear traction-separation response within the cohesive zone modelling scheme. Following the research in [23], the fatigue load was applied, as shown in Figure 3. At first, the maximum load (the tensile load applied to the joint) was delivered in a static step, where no accumulation of fatigue damage took place. The fatigue step came next, where the peak load was maintained and the fatigue deterioration was computed using the cohesive model assumption. The number of loading cycles during this cycle was assumed to be proportional to the time increment of the analysis [21]. 

### 2.2. Constitutive Equations

In the cohesive model used, the static and fatigue damages are calculated separately, and later they are added to each other to obtain the total damage. A bi-linear traction-separation law was used. The mixed-mode equivalent displacement jump $\lambda_{mix}$ was expressed in terms of the separation for the opening (Mode 1, λ*_normal_*) and the shear (Mode 2, λ*_shear_*) modes as below:

(1) $$\lambda_{mix}=\frac{K_{shear}\lambda_{shear}{}^{2}+K\lambda_{normal}{}^{2}}{\sqrt{K_{shear}{}^{2}\lambda_{shear}{}^{2}+K^{2}\lambda_{〈normal〉}{}^{2}}}$$

where $〈.〉$ is the Macaulay operator used to disregard the negative values, as it is assumed that a compressive loading characterized by a negative $\lambda_{normal}$ does not result in any damage [24]. K and $K_{shear}$ are the stiffnesses for the opening and shear modes, respectively. The mixed-mode onset displacement jump ($\lambda_{mix}^{0}$) and critical displacement jump ($\lambda_{mix,c}$) are as follows:

(2) $$\begin{array}{c} \lambda_{mix}^{0}=\sqrt{\frac{K〈\lambda_{normal}〉{}^{2}+\left[K_{shear}\lambda_{shear}{}^{2}-K{\left(\lambda_{normal}^{0}\right)}^{2}\right]{\left[B\right]}^{n}}{K_{B}}}\\ \lambda_{mix,c}=\frac{K\lambda_{normal}^{0}\lambda_{normal}^{c}+\left[K\lambda_{shear}^{0}\lambda_{shear}^{c}-K\lambda_{normal}^{0}\lambda_{normal}^{c}\right]{\left[B\right]}^{n}}{K_{B}\lambda^{0}} \end{array}$$

Here, $\lambda_{i}^{c}=2*G_{i,c}/\tau_{i,c}$, *i* = Modes 1 (normal) and 2 (shear) with $G_{i,c}$ and $\tau_{i,c}$ are the critical strain energy release rate and interfacial strengths, respectively. $K_{B}$ is the mode dependent penalty stiffness and is equal to $K\left(1-B\right)+K_{shear}$ with B as the local mixed-mode ratio [21,24]. *n* is the constant from the Benzeggagh–Kenane criterion [25]. Furthermore, the mixed-mode interlaminar strength is calculated as follows:

(3) $$\tau_{mix,c}{}^{2}=\tau_{normal,c}{}^{2}+\left(\tau_{shear,c}{}^{2}-\tau_{normal,c}{}^{2}\right)B^{n}$$

The static damage ($D_{static}^{t}$) at the current time step is calculated by the following:

(4) $$D_{static}^{t}=\frac{\lambda_{mix,c}\left(\lambda_{mix}-\lambda_{mix}^{0}\right)}{\lambda_{mix}\left(\lambda_{mix,c}-\lambda_{mix}^{0}\right)}$$

A critical damage parameter, i.e., the damage threshold $r^{t}$ is defined to check whether the displacement jump is big enough to yield damage in the model, as follows:

(5) $$\begin{array}{c} r^{t}=\frac{\lambda^{0}\lambda_{mix,c}}{\lambda_{mix,c}-D_{static}^{t}\left[\lambda_{mix,c}-\lambda^{0}\right]}\\ r^{t+1}=\max\left\{r^{t},\lambda_{mix}\right\} \end{array}$$

If the equivalent displacement jump exceeds the damage threshold, the static damage at the next step is calculated by:

(6) $$D_{static}^{t+1}=\frac{\lambda_{mix,c}\left(r^{t+1}-\lambda_{mix}^{0}\right)}{r^{t+1}\left(\lambda_{mix,c}-\lambda_{mix}^{0}\right)}$$

To calculate the damage due to the fatigue loading, the damage model in [24] was adapted. In the fatigue analysis, the force is kept constant after it reaches its maximum at the end of the static step. $\partial D_{i}/\partial N$ is the rate of fatigue damage progress at time step *i* and is calculated using:

(7) $$\frac{\partial D_{i}}{\partial N}=\frac{1}{l_{CZ}}\frac{{\left(\lambda_{mix,c}\left(1-D\right)+D\lambda_{mix}^{0}\right)}^{2}}{\lambda_{mix,c}\lambda_{mix}^{0}}\frac{da}{dN}$$

where $l_{CZ}$ is the length of the cohesive zone and is equal to $\left(9\pi /32\right)\left(E_{mix}G_{mix,c}/{\left(\tau_{mix,c}\right)}^{2}\right)$ with $E_{mix}$ is the mixed-mode of Young’s modulus [24].

The crack growth rate due to fatigue loading (da/dN) is calculated using the Paris law [26,27] $da/dN=C.\Delta G^{m}$, where C and *m* are the material constants. ΔG is the variation in the strain energy release rate within each fatigue cycle and is calculated by $\Delta G=G_{max}\left(1-R^{2}\right)$, where *R* is the load ratio, the ratio of the lowest and highest loads during the fatigue loading, and $G_{max}$ is characterized by the area under the traction separation curve, as in the following:

(8) $$G_{max}=\frac{\tau_{normal,c}}{2}\left[\lambda_{mix,c}-\frac{{\left(\lambda_{mix,c}-\lambda_{max}\right)}^{2}}{\left(\lambda_{mix,c}-\lambda_{mix}^{0}\right)}\right]$$

where $\lambda_{max}$ is the maximum displacement jump during the loading cycle. The following condition is necessary for the stable crack to spread: $G_{th}<G_{max}<G_{mix,c}$. This means that if the strain energy release rate is more than the critical strain energy release rate, $G_{mix,c}$, is calculated using the Benzegaggh–Kenane criterion. If it is less than the threshold value $G_{th}$, the crack cannot propagate in a stable mode. In accordance with [22,24] $0.01G_{mix,c}$ is chosen for $G_{th}$ as the cutoff value. 

In the computations, a cycle jump strategy [28] is utilized to avoid the lengthy computation time caused by the large number of cycles. The next shows how the damage variable at time step $i+\Delta N_{i}$ is determined.

(9) $$D_{i+\Delta N_{i}}=D_{i}+\frac{\partial D_{i}}{\partial N}\Delta N_{i}$$

where $D_{i}$ is the fatigue damage variable at time step *i*. $\Delta N_{i}$ is the number of cycles skipped before moving on to the next time step. It affects the precision of the results and is estimated using:

(10) $$\Delta N_{i}=\frac{\Delta D_{max}}{\frac{\partial D_{i}}{\partial N}}$$

Here, $\Delta D_{max}$ is the maximum damage increase and is chosen by the user for its smaller value leading to more accurate results. The value of 0.005 was chosen for the present study [29]. Eventually, the total damage is calculated as the summation of static and fatigue damages $D_{total}=D_{static}+D_{i+\Delta N_{i}}$. The constitutive equations of the cohesive zone model are taken into account in the computations via the UMAT subroutine. Their details and the flowchart of the subroutine can be found elsewhere [21,29].

The material parameters that were employed in the simulations to predict the behaviour of the adherends and the adhesive layer are shown in Table 1. Basically, they are the elastic modulus (*E*), Poisson’s ratio (ϑ), and yield strength ($\sigma_{Y}$) for the adherends and the interface stiffness (*K*); ϑ are the interfacial strengths for the normal and shear modes ($\tau_{i,c}$) and their critical strain energy release rates ($G_{i,c}$), and *n* is used to calculate the mixed-mode fracture toughness for the adhesive layer.

## 3. Results and Discussion

In this section, we discuss the experiments from the literature that were used to calibrate and validate the numerical model. The impacts of the different step-lap configurations on the service life of the joint were then carefully examined.

### 3.1. Validation of the FE Model

**Figure 4:**
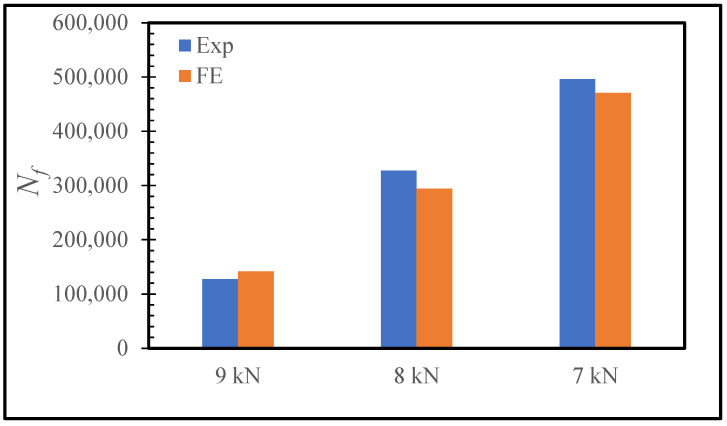
Number of cycles to failure (*N_f_*) for the three-step-lap joint subjected to different amounts of cyclic tensile loads obtained experimentally [15] and numerically.

For the calibration of the Paris law constants *C* and *m* (see Table 1), the three-step-lap joint subjected to a 9 kN fatigue stress was taken into consideration. A load ratio (R) of 0.1 was used. The failure cycle *N_f_* was one of the outcomes of the computations used to evaluate the performance of the joint, and it was used here to validate the developed FE model following the studies reported in [21,31,32]. Here, it was defined as the number of cycles at which the damage reached a minimum of 70% at all of the material points of the adhesive layer, as the simulations demonstrated that the lap joint became highly unstable upon reaching this damage distribution. For the values of *C* = 1.0 × 10^−12^ N/mm^3^ and *m* = 2.0, *N_f_* was found to be 141,850 cycles (see Figure 4). This was found to be in agreement with the experimentally obtained *N_f_* (127,566 cycles) in [15]. For verification purposes, the lap joint was also loaded with 8.0 kN and 7.0 kN loads. It was observed that *N_f_* was equal to 294,310 cycles and 470,830 cycles. They were in line with those obtained experimentally: 327,566 cycles and 495,884 cycles (Figure 4). Because the experimental results were simply the average of three experiments, and their variances were not included in [15], the difference between the experiments and FE results here, we believe, are acceptable. If the deviations had been included, a more accurate comparison might be made. The experimental results in [5] revealed that a lower fatigue load resulted in a longer fatigue life, and the modelling findings here support this observation. Our additional simulations demonstrated that *N_f_* reached more than 10^6^ cycles for 6 kN loading, and much higher cycles were attained for even lower loads. In [15,33], 10^6^ cycles were considered to be the fatigue lifetime of the lap joints. Moreover, 10^7^ lifetime is typically considered for the composite structures used in aviation applications. We believe that the applicability of the present model can be extended to the predictions for the fatigue performance of lap joints with the dimensions and configurations used in the aviation industry.

**Figure 5:**
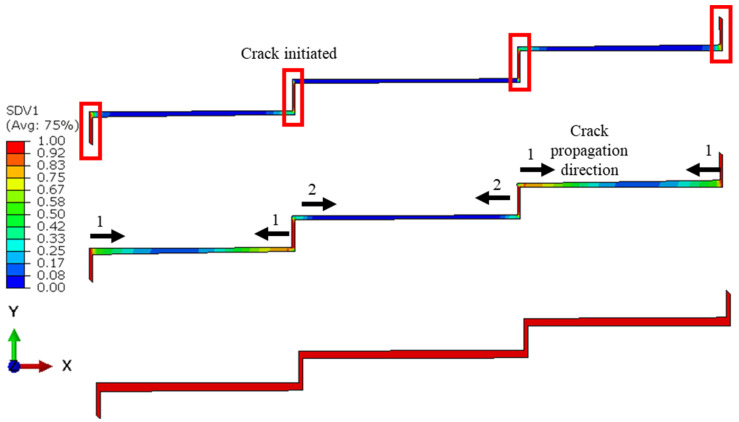
Distribution of the fatigue damage in the adhesive layer with the crack propagation direction and its sequence for the three-step-lap joint subjected to 8 kN tensile loading at *N_f_*/3 (**top**), 2*N_f_*/3 (**middle**), and when the complete damage occurred at all of the points of the adhesive layer (**bottom**). Numbers 1 and 2 in the figure represent the sequence of the crack propagation.

Figure 5 presents the distribution of the fatigue damage in the adhesive layer for the three-step-lap joint subjected to 8 kN tensile loading at *N_f_*/3, 2*N_f_*/3, and upon the complete damage reached in the adhesive layer. Firstly, it was observed that the damage was initiated and reached completion in the elements oriented in the vertical direction. As the adhesive elements in these cross-sections were exposed to higher normal stresses when compared to other cross-sections, and knowing that the adhesives were quite poor against the peel loads, the onset of damage occurred there. Secondly, the damage started to propagate in Region A (see Figure 1) followed by Region B.

### 3.2. Effect of the Number of Steps

**Figure 6:**
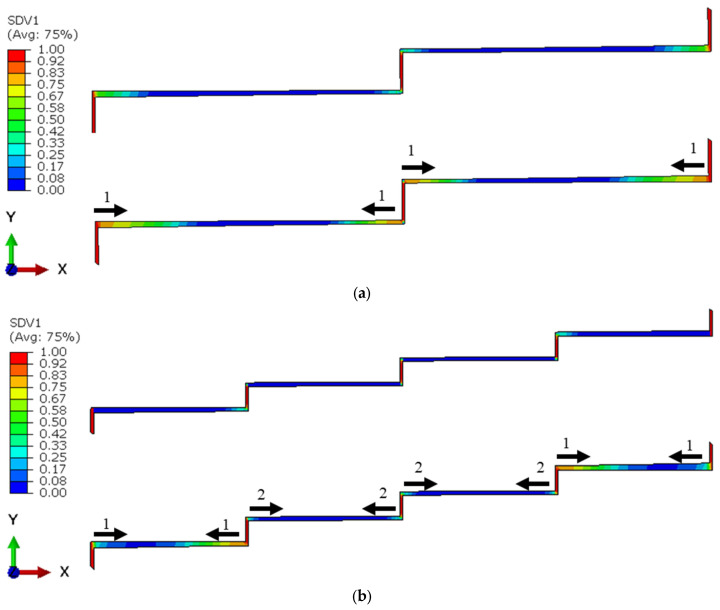
Distribution of the fatigue damage in the adhesive layer with the crack propagation direction and its sequence for the two- (**a**) and four-step-lap joints (**b**) subjected to 8 kN tensile loading at *N_f_*/3 (**top**) and 2*N_f_*/3 (**bottom**). Numbers 1 and 2 in the figure represent the sequence of the crack propagation.

This section looks into how the number of steps in the lap joint affect the SLJ’s fatigue response. To this end, the two-step- and four-step-lap joint configurations were analysed and compared with the three-step one when they were subjected to 8 kN load. Table 2 compares their *N_f_* values. They were 262,620, 294,310, and 342,130 cycles for the two-, three- and four-step-lap joints, respectively. It was observed that the service life of the joint increased with an increase in the number of steps. Figure 6 presents the distribution of the damage in the adhesive layer at *N_f_*/3 and 2*N_f_*/3 for the two- and four-step-lap joints. Similar to that of the three-step-lap joints, the damage started in the elements oriented in the vertical direction and later expanded in the horizontal direction. However, the damage propagation occurred more in the horizontally oriented elements closer to both ends of the step layer than those located towards the centre. As there were no central regions left in the adhesive layer where the crack could expand at a later stage for the two-step-lap joint (Region B does not exist, see Figure 2 and Figure 6a), the lap joint failed earlier. Moreover, in the three- or four-step-lap joints, the crack propagated in Region A first, then Region B (see Figure 1, Figure 2, Figure 5 and Figure 6b), where more cycles were required for the completion of the joint’s service life.

When the spread of the crack in Region A was compared with the two- and four-step-lap joints in Figure 6, it was noted that the crack expanded more symmetrically from the far left or right-end and mid-point of the of the adhesive layer for the former lap joint; however, the crack propagated more severely from the inner region when compared to the end side of the layer for the latter lap joint.

Considering the results presented in Table 2, one should keep in mind that the phenomenon of composite fatigue under cyclic loading is random in nature. There are different approaches presented in the literature [20] that aim to overcome the randomness of failure and propose different approaches, such as the random variable, to predict failure probability. These methods try to derive the probability of failure using probabilistic static damage curves for the matrix crack, delamination, and fibre breakage. In the current paper, the authors did not account for a random factor, therefore, the presented numbers of cycles should be considered as approximate values and interpreted as a value of order. (i.e., 416,940 cycles would imply that failure should be expected around 400k repetitive loadings.)

For the designs of adhesively bonded lap joints, it is crucial to understand how the cycle load causes cracks to form, i.e., crack initiation, in the SLJs. At lower loadings, this phase frequently dominates the fatigue life. Table 2 presents the damage initiation cycle (*N_i_*) and its ratio in percentage with respect to *N_f_* for the respective configurations. *N_i_* was determined by counting the cycles at which the damage at one end of the adhesive layer reached at least 90%, i.e., the damage started at a material point with substantial damage there. Cycles in the amounts of 127,420, 166,310, and 198,930 were noted for the onset of damage in the joints when the number of steps were increased from two to four in a row, respectively. Their respective *N_i_*/*N_f_* ratios were 48.50, 56.51, and 58.14%. It was concluded that the damage initiation in the step-lap joint was delayed with the increase in the step number.

**Figure 7:**
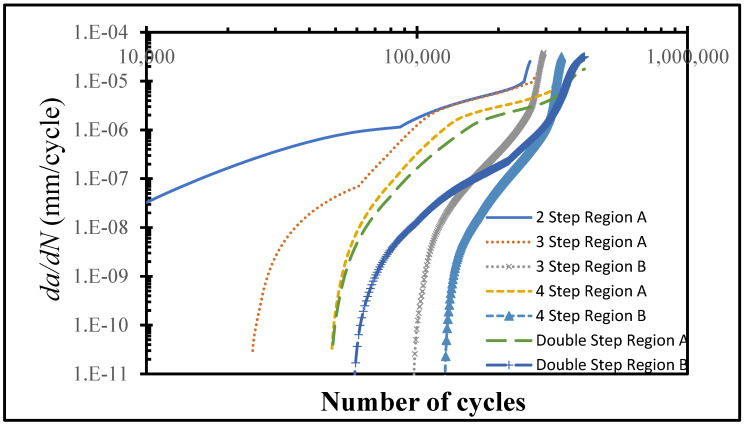
*da*/*dN* vs. the number of cycles at different parts of the adhesive layer of various lap joints with different step configurations and subjected to 8.0 kN cyclic tensile load.

*da*/*dN* plots were made for all of the configurations under consideration in order to obtain insight into the step-lap joints’ failure response, with respect to the number of steps (Figure 7). Overall, as the cyclic loading progressed, the rate of crack propagation increased, but they were either in an increasing or a decreasing manner. It was noticed that the two-step-lap joint had the largest *da*/*dN* in the course of the fatigue loading, proving the shortest *N_f_*, as seen in Table 2. A sudden jump was noticed when *N* = 246,620 cycles. At this number of cycles, the damage reached all of the elements, especially those at the mid-region of the adhesive layer, where, onwards, the crack propagation occurred very quickly. When the three-step-lap joint was analysed, the crack propagation was observed to begin in Region A at around the 24,000th cycle (see Figure 1), which was much earlier than in Region B, where it started around the 97,000th cycle. It should be emphasized that at these cycles, the damage just began at a material point with a small amount of damage and should not be mixed with *N_i_*. It was noted that while the propagation rate was more in a saturation mode in Region A (except for the last part of the loading), it was in a steeper mode in Region B. That explains how the *da*/*dN* for Region B reached the 1.00 × 10^−5^ mm/cycle, which is similar to that reached in Region A before the fatigue failure, even though the crack propagation in Region B started at a much later stage. A similar distribution of *da*/*dN* was attained for the four-step-lap joint. The main difference was the number of cycles at which the crack started to propagate in different regions. For instance, the crack began to expand at 48,000 cycles and 126,000 cycles in Regions A and B, respectively, with a difference of 78,000 cycles for the lap joints with four steps, while this difference was 73,000 cycles (24,000, 97,000 cycles) for the one with three steps. When the *da*/*dN* values of the lap joints were compared for the three- and four-step-lap joints, they were smaller for the latter one. That proved the longer service life of the step-lap joint with a greater number of steps. The *da*/*dN* plots of the step-lap joints differed from those of the single-lap joints (see [21]), where in the latter, the crack growth rate increased gradually over the course of the fatigue loading, followed by a sudden jump just before the fatigue failure. However, in the step-lap joints, as different parts of the adhesive layer at different steps affected each other during the loading, the *da*/*dN* curves showed different characteristics.

### 3.3. Influence of the Configuration: Double Stepping

In this section, the influence of the double-step configuration on the performance of a lap joint was investigated. Its configuration was given in Figure 2. In fact, its configuration was adapted from four-step-lap joint, where the x-axis, located at mid-height of the adhesive layer, was taken as the mirror line for a half-length of the adhesive layer. Table 2 shows that its *N_f_* is 416,940, which is a 21.8% longer service life than that of its four-step counterpart. Moreover, as its *N_i_* was 212,940, the *N_i_*/*N_f_* value was calculated to be 51.07%. Comparing this with the *N_i_*/*N_f_* value of the four-step one (58.14%), it was concluded that when the double-step was used, the damage was initiated in the adhesive layer earlier, but the fatigue failure occurred at a later stage.

**Figure 8:**
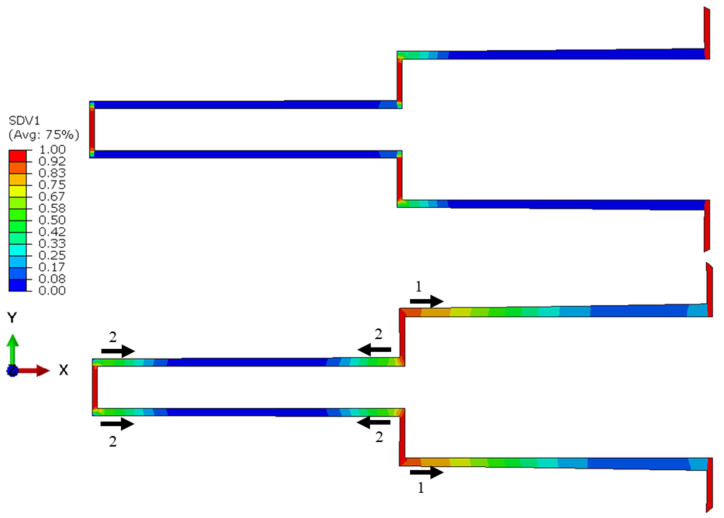
Distribution of the fatigue damage in the adhesive layer with the crack propagation direction and its sequence for the double-step-lap joint subjected to 8 kN tensile loading at *N_f_*/3 (**top**) and 2*N_f_*/3 (**bottom**). Numbers 1 and 2 in the figure represent the sequence of the crack propagation.

Figure 8 presents the distribution of the fatigue damage in the adhesive layer for the double-step-lap joint under 8 kN tensile loading at *N_f_*/3 and 2*N_f_*/3. It was observed that the crack was initiated in Region A first, then in Region B; while the crack propagated mostly only from the left side of the adhesive layer in the first region, it was propagated from both ends symmetrically in the second region. This could be seen also from Figure 7 that shows the *da*/*dN* vs. the number of cycles. While the onset of the crack in Region A was at the 48,000th cycle, it was at the 58,000th cycle in Region B with a cyclic distance of 10,000 cycles. Unlike the two-, three-, and four-step-lap joints, the crack initiated and propagated in different regions more simultaneously for the double-step-lap joint.

## 4. Concluding Remarks

This study investigated the fatigue performance of various configurations of step-lap joints subjected to tensile loading. For this purpose, their advanced FE models were developed, where the fatigue damage model was integrated into the cohesive zone model to describe the behaviour of the adhesive layer using UMAT subroutine. The model was successfully validated using experiments from the literature.

The findings were as follows:

- The service life of step-lap joints increased with an increase in the number of steps;
- The onset of damage in the adhesive layer of the step-lap joint was delayed with an increase in the step number, where the crack growth rate also became smaller;
- The characteristics of *da*/*dN* curve for the outer steps of the adhesive layer were different from those of the inner steps;
- The lifetime of the double-step-lap joint was 21.8% longer than that of its counterpart, the four-step-lap joint.

## Figures and Tables

**Table 1:** The material parameters for AA2024-T3 and DP460 used in the FE simulations [21,30].

AA2024-T3	*E* (MPa)	ϑ	$\sigma_{Y}$ (MPa)				
	72,400	0.33	324				
DP460	*K* (N/mm^3^)	ϑ	$\tau_{i,c}$, *i* = *normal*, *shear* (MPa)	$G_{i,c}$, *i* = *normal*, *shear* (N/mm)	C (N/mm^3^)	*m*	*n*
	10^14^	0.38	32.6, 28.5	2.56, 11.71	10^−12^	2.0	2.1

**Table 2:** *N_i_* and *N_f_* and their ratios (*N_i_*/*N_f_*) in percentage for various step-lap joints.

Load (N), t (mm)	Two Step	Three Step	Four Step	Double Step
*N_i_*	127,420	166,310	198,930	212,940
*N_f_*	262,620	294,310	342,130	416,940
*N_i_*/*N_f_* (%)	48.50	56.51	58.14	51.07

## Data Availability

The data presented in this study are available on request from the corresponding author.
